# Isolation and characterization of four novel *Vibrio parahaemolyticus* bacteriophages from oysters

**DOI:** 10.1371/journal.pone.0339894

**Published:** 2025-12-29

**Authors:** Joud Aldaroub, Chrissy M. Walsky, Rylee E. Ewell, Frank O. Aylward, Ann M. Stevens, Alison Kernell Burke

**Affiliations:** 1 Department of Biological Sciences, Virginia Tech, Blacksburg, Virginia, United States of America; 2 Center for Emerging, Zoonotic, and Arthropod-borne and Pathogens, Virginia Tech, Blacksburg, Virginia, United States of America; 3 Department of Biology, Virginia Military Institute, Lexington, Virginia, United States of America; Bigelow Laboratory for Ocean Sciences, UNITED STATES OF AMERICA

## Abstract

*Vibrio parahaemolyticus* (VP) is a bacterial pathogen found in brackish and marine water that infects many marine organisms, such as oysters and shrimp. Consumption of raw or undercooked seafood contaminated with *V. parahaemolyticus* is a primary cause of seafood-borne gastroenteritis in humans. Due to increasing ocean temperatures, *V. parahaemolyticus* contamination of oyster beds in the United States has spread up the east and west coasts to the northern-most states. Promising new research is exploring the isolation of bacteriophages against *V. parahaemolyticus* with a long-term goal to possibly decontaminate oyster beds, thereby expanding the harvest season and allowing for safer consumption of seafood. In this study, store-bought oysters harvested from the Chesapeake Bay in Virginia were used to isolate four bacteriophages with activity against a specific *V. parahaemolyticus* strain. A standard double agar overlay plaque assay was used to identify phage activity. After phage isolation, the genomes were sequenced, and transmission electron microscopy (TEM) was performed to visualize the virions. The genomes and TEM images revealed four distinct phages. Three of the phages are distinct isolates that exhibit podovirus-like morphology with short tails and genome sizes of approximately 43 kbp. One phage has siphovirus-like morphology and is a mid-sized tailed phage with a genome size of 80 kbp. Although spot tests performed with the oyster homogenates on up to 10 different *V. parahaemolyticus* strains recovered activity across a wide range of hosts, plaque assays with the isolated phages showed limited host range. Future work will be necessary to determine the viability of using the bacteriophages for elimination of *V. parahaemolyticus* in harvested oysters, treatment of aquaculture seed and spat, and/or the environment.

## Introduction

*Vibrio parahaemolyticus* is a Gram-negative halophilic bacterium widely found in estuarine, marine, and coastal surroundings [1]. It exhibits a versatile lifestyle, existing either as a free-swimming organism, an inhabitant attached to underwater surfaces or associated with different shellfish species. Since its discovery in 1950, *V. parahaemolyticus* has become a leading cause of seafood-borne illness worldwide [2]. Many strains are pathogenic to humans and can cause diseases including wound infections, septicemia and most commonly acute gastroenteritis, which is contracted through the ingestion of raw or undercooked seafood, specifically shellfish [3]. Beyond human health implications, *V. parahaemolyticus* is responsible for a significant impact with substantial economic losses in the aquaculture industry causing harm to fish, shrimp and oyster hatcheries [4].

A primary reservoir of *V. parahaemolyticus* is the Eastern oyster (*Crassostrea virginica*), where bacterial abundance peaks during the warmer summer months. Adult oysters, as filter feeders, can filter up to 50 gallons of seawater per day, leading to bacterial concentrations in their tissues that can be up to 100 times greater than in the water column [5]. This accumulation poses a significant public health risk, as raw or undercooked oysters are a primary vehicle for *V. parahaemolyticus* transmission. Seasonal warming, especially in the Chesapeake Bay, further elevates *V. parahaemolyticus* abundance, increasing public health risks during peak oyster harvesting periods. However, Eastern oysters also play a crucial ecological role as keystone species by enhancing water quality and providing essential habitat for marine life [6]. The Chesapeake Bay, a major estuary in the United States and a vital source of commercial shellfish, has experienced fluctuating oyster populations due to overharvesting, habitat destruction, and disease. The native oyster population declined to less than 1% of its historical levels from the late 1800s, prompting conservation and restoration efforts [7]. Restoration efforts in the Chesapeake Bay have emphasized oyster repopulation through hatchery-based aquaculture. The Chesapeake Bay Watershed Agreement, signed in 2014, established a goal of restoring the region’s oyster population by introducing over 10 billion new oysters into the bay and ten of its tributaries through aquaculture-based initiatives [8].

The persistence of *V. parahaemolyticus* in oysters poses a significant public health risk, particularly during summer months when the bacteria proliferate due to increased water temperatures. Traditional mitigation strategies, such as post-harvest processing or refrigeration, offer limited success in controlling *V. parahaemolyticus* [9]. Moreover, the use of antibiotics is prohibited in U.S. bivalve aquaculture due to regulatory concerns over resistance development and environmental impact [10]. As a result, there is growing interest in exploring alternative strategies, including the use of bacteriophages as natural biocontrol agents.

Bacteriophages, or phages, are viruses that specifically infect and lyse bacterial cells, offering a targeted and environmentally sustainable approach to bacterial growth suppression. Recent studies have highlighted the potential of phages to control *V. parahaemolyticus* in seafood. For example, the novel bacteriophage vB_VpaS_PG07 has demonstrated broad-host-range activity, significantly reducing bacterial mortality in shrimp models [11]. Similarly, the phage vB_VpaS_OMN, isolated from oysters, effectively decontaminated *V. parahaemolyticus* from seafood surfaces, underscoring its potential as a biocontrol agent [12]. The application of phages in aquaculture has been explored through various methods, including direct water treatments, oral administration via feed, and injection [13]. Notably, the jumbo *Vibrio* bacteriophage PVA8 has exhibited promising antibacterial properties such as broad host range, large burst size, and decent tolerance to adverse conditions, reducing *V. parahaemolyticus* levels in aquaculture and improving shrimp survival rates from 34.43% to 88.89% [14]. These findings emphasize the potential of phage therapy in managing bacterial infections and enhancing seafood safety for oysters.

The identification and characterization of novel bacteriophages from oysters targeting *V. parahaemolyticus* also provides valuable insights into their biological diversity. Prior research has described various *Vibrio* phages with unique morphological and genomic traits, demonstrating their adaptability and efficiency in bacterial suppression [15,16]. A critical gap exists in characterizing naturally occurring phages from endemic environments like the Chesapeake Bay, especially those associated with oysters. Such studies are essential for identifying phages adapted to local *Vibrio* strains and environmental conditions. Furthermore, little is known about the diversity, genomic content, and host specificity of oyster-associated *V. parahaemolyticus* phages.

This study aimed to isolate and characterize novel bacteriophages from oysters harvested in the Chesapeake Bay, focusing on their efficacy in targeting *V. parahaemolyticus*. By identifying naturally occurring bacteriophages within this ecosystem, we seek to expand our understanding of their role in mitigating bacterial populations in oysters and identify phages for potential future application as biocontrol agents in aquaculture and seafood safety management.

## Materials and methods

### Bacterial strains and growth conditions

A total of 10 *V. parahaemolyticus* strains listed in Table 1 were used for testing the oyster homogenate for phage activity. *V. parahaemolyticus* strain G12408 served as the host for enrichment and plaque isolation. All *V. parahaemolyticus* strains were grown less than 18 hours in Tryptic Soy Broth (TSB) with 2% NaCl at 37ᵒC in a shaker at 200 rpm. The 18-hour culture was diluted in fresh TSB with 2% NaCl to an OD_600_ of 0.05 and grown at 37ᵒC in a shaker at 250 rpm to an OD_600_ of 0.4–0.6 to be used in enrichment or isolation protocols.

**Table 1:** Strains of *Vibrio parahaemolyticus* used in the host range determination.

	Known Strain Information			
VP Strains	Isolation Information	Sequence Type	Serotype	Reference
CT4291	Environmental isolate	674		[17]
G12408	Obtained from the Oyster River tributary of the New Hampshire Great Bay Estuary in 2019	2020		C. Whistler
G13119	Obtained from an oyster in the Oyster River tributary of the New Hampshire Great Bay Estuary in 2020	400		C. Whistler
LM5312	BB22 OP strain of an environmental isolate from Bangladesh in 1980		O4:K8	[18]
MAVP-26	Clinical isolate-Pacific oyster borne gastroenteritis 2013	36	O4:K12	[19]
MAVP-K	Clinical isolate from 2011	8		[20]
RIMD2210633	Clinical isolate from Japan in 1996	3	O3:K6	[21]
VIB373	FDA acquired strain isolated from oysters			FDA
VIB374	FDA acquired strain isolated from oysters			FDA
VIB389	FDA acquired strain isolated from a clinical sample			FDA

### Oyster acquisition and handling

The live consumer-ready oysters that had been harvested from the Chesapeake Bay in VA were purchased from a local grocery store and used as the source for phage isolation. The newly isolated phages were named for the oyster they were isolated from or the initials of the researchers involved in the isolation. Oysters do not require IACUC approval for research but were either immediately shucked and homogenized or handled using the best management practice with optimal water quality and aeration to keep them alive during bacterial enrichment. After enrichment they were either frozen or shucked for homogenization. The oysters were purchased during the summer because it has previously been shown that there are higher levels of *V. parahaemolyticus* in the oysters during warmer months in the United States [22]

### Phage enrichment for OYD and SMILEY

Two oysters, OYD and SMILEY and were acclimated to room temperature in a cooler with 20 ppt (g/l synthetic sea salt) artificial seawater (Crystal Sea Marinemix, Marine Enterprises International, Baltimore, MD) before placement in separate beakers. On day 2 they were inoculated with ∼10^6^–10^7^ CFU/ml final concentration of *V. parahaemolyticus* strain G12408 in the water column for SMILEY and *V. parahaemolyticus* strain RIMD2210633 in the water column for OYD following the exposure protocol described by Hines et al 2022 [22]. After an extended bacterial enrichment, the whole oysters were frozen until shucked and homogenized with 100% ethanol-sanitized OmniTip soft tissue probes attached to an OmniTip homogenizer (Omni International, Kennesaw, GA) at day 6. In contrast, CREW and REJA were immediately shucked without any bacterial enrichment.

### Phage isolation, preparation, and propagation

The whole oyster was homogenized in a 50 mL conical tube and SM buffer (100 mM NaCl, 8 mM MgSO₄·7H₂O, 50 mM Tris-HCl, 0.01% gelatin) was added to bring the volume to 35 mL. The homogenized oyster tissue for OYD and SMILEY in SM buffer was incubated at room temperature for 1 hr. The homogenate for CREW in SM buffer was incubated at 30°C for 1 hr with shaking at 250 rpm. The homogenates were then subjected to 2,000 × g for 10 min in an Avanti J-26 XP centrifuge equipped with a JS-5.3 swinging-bucket rotor (Beckman Coulter, Brea, CA, USA). OYD, SMILEY and CREW were isolated from supernatants that were filtered using a 0.45 μm syringe filter, while a 0.7 μm syringe filter was used in the isolation of REJA. The resulting filtrates were stored at 4°C overnight.

For enrichment of CREW, 0.5 mL of filtrate from the CREW oyster was inoculated with 0.5 mL of *V. parahaemolyticus* strain G12408 (OD_600_ = 0.5) and incubated at 30°C for 2 days with shaking at 220 rpm. The sample was then subjected to centrifugation twice at 10,000 rcf for 1 min and the supernatant was transferred to a new tube and stored at 4 °C for 5 days. For the isolation of CREW, OYD, and SMILEY, spot tests were performed with the filtered whole oyster homogenate against ten *V. parahaemolyticus* strains (Table 1). Briefly, 100 μL of each bacterial strain in mid-log phase (OD_600_ = 0.4) was added to 4.5 ml of molten Luria-Bertani (LB) (5 g tryptone, 12.5 g NaCl, and 2.5 g yeast extract) + 2% NaCl soft top agar (0.4% agar) and poured onto the surface of solidified LB bottom agar (2% agar). Then, 10 μl of each of the oyster homogenate was spotted onto the lawn and incubated at 32°C overnight. Finally, the results were analyzed with a positive zone of clearing indicating a positive spot test result and no zone of clearing indicating a negative result.

A flamed sterilized spatula was used to harvest the clearest plaques created in the soft agar and the plaque was placed into 4 mL of SM buffer and stored at 4 °C overnight. These plaques potentially represent multiple different phages in the oyster homogenate. Therefore, three rounds of plaque assays were completed using *V. parahaemolyticus* strain G12408 for isolation of individual phages, as described by [23].

For the isolation of REJA, the oyster homogenate was incubated with 500 μL of mid-log phase *Vibrio parahaemolyticus* G12408 for 10 min at room temperature. The homogenate was then serially diluted and ten μl of each 10-fold dilution was added to 4 mL of LB + 2% NaCl molten soft top agar (0.4% agar) and poured onto solidified 0.4% LB + 2% NaCl bottom agar (1.5% agar). Plates were incubated overnight at 37°C. Isolated plaques were picked with a sterile pipette tip and re-plated for three rounds to purify individual phages using the double-layer agar method, with *V. parahaemolyticus* strain G12408 serving as the phage host, as described by [23]. Host range determination for REJA was performed via spot tests as described above for the isolation of SMILEY, OYD, and CREW.

### High titer phage stock

The SEA PHAGES protocol was used to create high-titer phage lysates [24]. Briefly webbed plates (confluent plaques over a bacterial lawn) were flooded with 6 ml of SM buffer and incubated for 1 hour at room temperature. Lysate was collected with a sterile syringe and subjected to centrifugation at 5000 rpm for 7 min. The supernatant was then filtered with a 0.45 μm syringe filter. Phage particles were precipitated using 20% polyethylene glycol (PEG-8000) and 2.5 M NaCl at 4°C overnight. Samples were processed at 10,000 × g for 20 min, at 4° C using an Avanti J-26 XP centrifuge equipped with a JA-20 fixed-angle rotor (Beckman Coulter, Brea, CA, USA) and the resulting pellets were resuspended in SM buffer. A vortex was used to resuspend the pellets for CREW, OYD, and SMILEY but not REJA. An equal volume of chloroform was added to lyse remaining bacterial cells. The titer of the high phage stocks was calculated using serial dilutions plaque assays as follow: REJA had a titer of 1.04 x 10^11^ pfu/ml, OYD had a titer of 2.15 x 10^10^ pfu/ml, CREW had a titer of 1.78 x 10^10^ pfu/ml and SMILEY had a titer of 6 x 10^10^ pfu/ml. Lysates were stored at 4°C for future work.

### Transmission electron microscopy

Phage morphology was examined using transmission electron microscopy. Five microliters of high-titer phage lysate were placed onto 300-mesh copper grids (Ted Pella, Inc.) and negatively stained with 4% (w/v) uranyl acetate (pH 7.2). Samples were imaged using a JEM-2100 Transmission Electron Microscope (JEOL, Tokyo, Japan) at the Corporate Research Center, Virginia Tech.

### DNA extraction and sequencing

Phage DNA was extracted using the Promega Wizard DNA Clean-Up Kit following the manufacturer’s protocol. The DNA quality and concentration were assessed using a Qubit Fluorometer (Invitrogen, Thermo Fisher Scientific, Waltham, MA, USA) before next-generation sequencing was performed with the Illumina MiSeq sequencing platform using the NEB Ultra II Library Kit and 100 bp single-end reads at the North Carolina State University Genomic Sciences Laboratory.

### Computational analysis and genomic comparison

The NCSU Genomic Sciences Lab used QIAGEN CLC Genomics Workbench 23.0 (QIAGEN, Aarhus, Denmark) to assemble the genomes of CREW, OYD, and SMILEY. The REJA genome was assembled using Newbler v2.9. The protein prediction was done using Prodigal v. 2.6.3 [25]. Annotations of the predicted proteins were completed using, HMMER3 v.3.4 [26] (an e-value of 1e-5 was used as a cut off), eggNOG Database [27], Virus Orthologous Groups Database (VOGDB) [28] and Protein family database (Pfam) [29] to ensure a precise prediction. A whole genome similarity dendrogram was constructed using the viral proteomic tree server (ViPtree) using fasta amino acid (.faa) files for each of the four phages individually and compared together [30]

### Accession numbers

GenBank accession numbers associated with this study are: CREW PX056653, OYD PX056654, SMILEY PX056655, and REJA PX056656.

## Results

### Host range and morphological characteristics of four phages infecting *V. parahaemolyticus*

Four bacteriophages infecting *V. parahaemolyticus* were isolated from store-bought oysters harvested from the Chesapeake Bay in VA, using spot tests and plaque assays after various enrichment methods were carried out (see Methods). The oyster homogenate that CREW was isolated from produced a uniform zone with complete clearing of the *V. parahaemolyticus* G12408 strain, partial clearing of the RIMD and CT4291 strains and faint clearing of the FDA strains VIB373 and VIB389. The spot test for the G12408 strain was used for isolation of CREW (S1 Fig) because it showed the most complete clearing. In contrast, the oyster homogenate that OYD and SMILEY were isolated from, produced spots with a halo of incomplete clearing around the cleared zone on plates seeded with *V. parahaemolyticus* strain G12408 (S2 Fig). The presence of halos around the zones of clearing suggests either a possible lysogenic phase or the potential production of depolymerases, which could enhance bacterial lysis by degrading biofilms or extracellular polysaccharides. There was no clearing observed on any of the other spot plates for each of the other nine *V. parahaemolyticus* strains. REJA was identified due to its formation of the smallest plaques produced on plates seeded with *V. parahaemolyticus* strain G12408 after 18 hours of incubation with a serial dilution of the filtered phage homogenate (S3 Fig).

**Fig 1:**
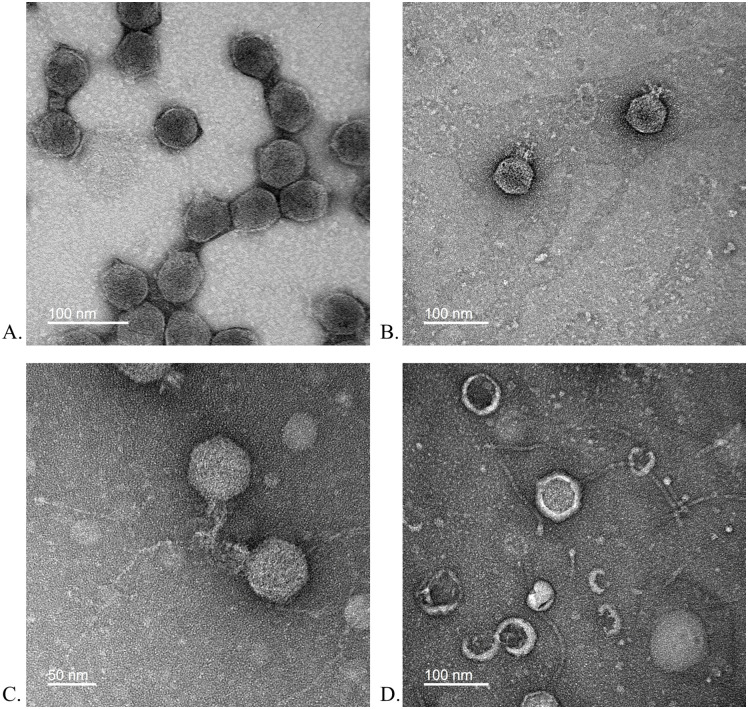
Transmission electron micrograph of *Vibrio parahaemolyticus* bacteriophages. **A.** CREW, **B.** OYD, **C.** SMILEY and **D.** REJA, shown with negative staining. Scale bars represent 50 or 100 nm as noted on image.

Transmission electron microscopy (TEM) analysis revealed that CREW, OYD and SMILEY exhibited podoviruses morphology with short tails, while REJA exhibited siphovirus-like morphology with longer tails. All four phage heads were similar in size (40 nm capsid diameters, Fig 1). Although some of the oyster homogenates showed a wide range of phage activity, after isolation plaque assay results demonstrated that all four purified phages only formed plaques on the environmental *V. parahaemolyticus* G12408 strain.

### Genomic analysis and protein prediction

The genomes of all four phages were sequenced using short-read Illumina technology and assembled. The assembled genomes were analyzed for predicted open reading frames (ORFs) and various programs were used to compare the predicted ORFs to known protein databases to try to identify associated functions (Table 2). CREW and OYD had similar genome sizes of 43,581 bp and 43,562 bp, respectively. SMILEY had a genome size of 47,155 bp, and REJA had a significantly larger genome of 80,495 bp. CREW had 66 predicted proteins, of which 51 had detectable homology to protein families in the VOG database, OYD had 65 predicted proteins with 51 VOG homologs, SMILEY had 45 predicted proteins with 13 VOG homologs proteins, and REJA had 99 predicted proteins with 32 VOG homologs.

**Table 2:** Genome and predicted protein comparison of the four bacteriophages.

Phage	Length (bp)	%GC	# of Predicted ORFs	eggNOG Annotated Proteins	VOG Identified Proteins	Pfam Predicted Proteins
SMILEY	47,155	45.9	45	0	13	16
CREW	43,581	43.8	66	15	51	36
OYD	43,562	43.8	65	15	50	33
REJA	80,495	46.2	99	0	32	16

### Phylogenetic analysis and relatedness

**Fig 2:**
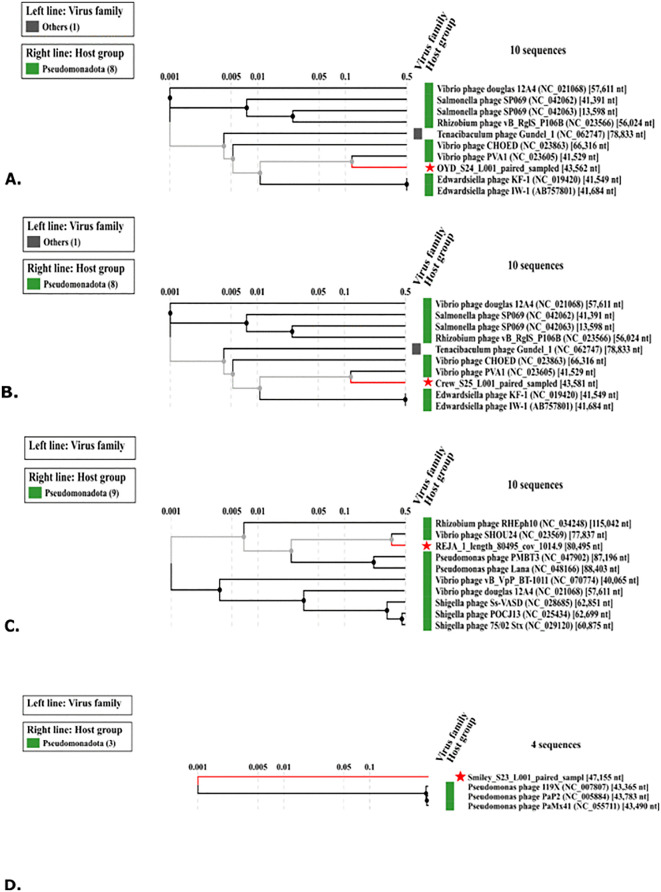
Whole genome similarity dendrograms of the four phages and their 10 close relatives created using ViP Tree. Dendrogram A is for OYD, B. is CREW, C. is REJA, and D is SMILEY. The dendrograms A, B and C are representative and not complete trees for the associated phages.

A whole-genome proteomic similarity dendrogram was constructed using the ViPTree database to assess evolutionary relationships for the phages. The 10 closest related sequences were used to generate the representative dendrogram for each phage (Fig 2), with the exception of SMILEY, for which only four related phages could be detected in the VipTree database. OYD and CREW each clustered most closely with *Vibrio* phage PVA1 showing sequence similarity and generating independent trees that each included 825 sequences from the ViPTree database. SMILEY clustered with *Pseudomonas* phages 119X, PaP2, PaMx41. While REJA clustered with *Vibrio* phage SHOU24 and was in the same clade as other *Pseudomonas* phages. The dendrogram generated by ViPTree included 174 sequences from the database that showed protein similarity.

**Fig 3:**
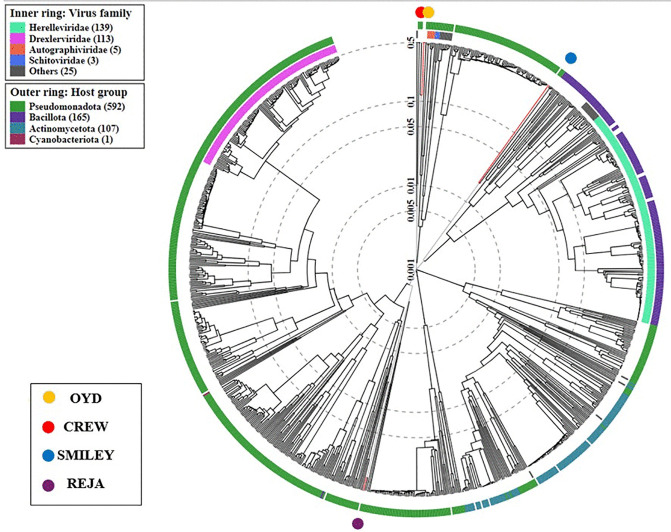
Whole genome similarity dendrograms. Dendrogram tree showing the distribution of OYD, CREW, SMILEY and REJA with a reference phage genome dataset. The tree was constructed using the VipTree server.

ViPTree was used to create a whole genome similarity dendrogram for comparing the four phages and how closely related they are to each other (Fig 3). All four phages belong to the *Caudoviricetes* class with OYD and CREW likely being distinct isolates of the same species in the genus *Lederbergvirus* because they show 99% similarity in their genomes. Whereas SMILEY clustered in a clade with *Pseudomonad* phages and REJA was the least related of the four phages and clustered in distant clade likely due to its larger genome size.

## Discussion

Bacteriophages have emerged as promising biocontrol agents for *Vibrio parahaemolyticus*, a major seafood-borne pathogen [31]. The four phages isolated in this study—CREW, OYD, SMILEY, and REJA—demonstrate genetic diversity and distinct morphologies, which could influence their efficacy in reducing *V. parahaemolyticus* contamination in oysters. Interestingly, the oyster that did not undergo extended enrichment steps prior to homogenization showed phage activity to a broader range of *V. parahaemolyticus* strains than when extended enrichment was performed with a specific strain prior to isolation. This finding has been previously observed, but without a clear explanation [32]. It is possible that the enrichment process is more effective at recovering phages active against the specific host strain used. The protocols used for finding phages against specific strains versus developing a phage cocktail active against multiple strains should be further explored.

SMILEY stands out among the four phages due to its unique genomic and phylogenetic characteristics. Unlike the other phages, SMILEY did not cluster with any previously characterized *Vibrio* phages in the ViPTree analysis but instead grouped with *Pseudomonas* phages, including 119X, PaP2, and PaMx41. Phage 119X was isolated from Australia, PaP2 was isolated in China, and PaMx41 was isolated from sewage in Mexico. All phages that clustered with SMILEY in the ViPTree analysis exhibited podovirus-like morphology [33]. This suggests that SMILEY may have evolved from phages that infect *Pseudomonas* or potentially acquired genes from pseudomonad phages through horizontal gene transfer events. Its podovirus-like morphology and 47,155 bp genome further distinguish it from the siphovirus-like phage REJA. Additional functional analyses performed on SMILEY, such as host adsorption assays and structural protein modeling, could provide further insight into its infectivity and potential cross-host interactions.

OYD and CREW clustered closely in the phylogenetic analysis with *Vibrio* phage PVA1 and had similar genome sizes (43,562 bp and 43,581 bp, respectively). They shared 99% identity, but are two distinct isolates of the same species with key genomic and functional differences. They were isolated from two different oysters, one that underwent extended enrichment and one that did not. Phage PVA1 was isolated from sewage samples obtained from local aquatic markets and morphological analysis revealed PVA1 has podovirus-like morphology [34]. CREW, isolated from the oyster homogenate that exhibited a broader host range, successfully infected multiple *V. parahaemolyticus* strains, while the OYD oyster homogenate OYD was restricted to a single strain (G12408). Functional predictions of the encoded proteins identified several protein differences between these two phages, which may contribute to their differential host specificity. CREW encoded an additional E3 ubiquitin-protein ligase RNF180 C-terminus (PF19332), an extra unidentified hypothetical protein, and a facilitator of iron transport (PF17357), these features were absent in OYD. Conversely, OYD encoded unique proteins, including a haemopoietic lineage transmembrane helix (PF15062), bacteriophage P22 NinX (PF10765), and a pre-mRNA splicing factor of the RES complex (PF09736), none of which were found in CREW. These differences could influence their infection mechanisms and lead to the conclusion that they are distinct phages.

REJA had the largest genome among the four phages (80,495 bp) and was also the most phylogenetically distant, clustering with *Vibrio* phage SHOU24, a lytic siphovirus. Previously characterized *Vibrio* phage SHOU24 was isolated from aquatic market sewage. It can infect *V. parahaemolyticus* strains containing the *tdh* genes and has a linear genome of 77,837 bp and GC content of 46% [35]. Larger phage genomes often encode additional auxiliary metabolic genes, structural variations, or unique lytic enzymes that could enhance their ability to infect and lyse bacteria more efficiently [36,37]. The greater number of predicted proteins (99 ORFs) in REJA compared to the other phages suggests potential functional advantages, such as resistance to bacterial defense mechanisms, or improved stability in environmental conditions.

Defining the characteristics of isolated phages is crucial for developing targeted phage therapies that effectively mitigate pathogenic *V. parahaemolyticus* strains while preserving the oyster microbiome [38]. The health and growth of oysters is dependent on their microbiome including resident bacteria and probiotic strains added to facilitate oyster development by protecting the growing seed and spat from infection [39]. Research has shown that altering bacterial communities in oysters can negatively impact their development and immune response. Not all oyster-associated *V. parahaemolyticus* strains are associated with human disease and should be targeted for decontamination. A targeted phage cocktail approach—combining phages with different host ranges to specific pathogenic *Vibrios*—could enhance specificity while reducing the risk of resistance development or damage to the oyster [40]. Therefore, phage therapy should be attempted in a manner that will not disrupt the natural microbiome of the oyster or probiotics used to help protect the oyster.

In addition to the use of broad host range phages or phage cocktails, slow-acting temperate (lysogenic) phages may have advantages in *V. parahaemolyticus* decontamination efforts, as they can integrate into bacterial genomes and may provide long-term suppression of the bacterial populations. However, some temperate phages can enhance the survival or virulence of their hosts, and their potential for lytic conversion and horizontal gene transfer requires careful evaluation before implementation [41]. Careful screening is therefore needed before temperate phages are used more broadly for phage therapy or biocontrol purposes. Given restrictions on antibiotic use in aquaculture, due to regulations regarding the maximum residue levels of antibiotics in the shellfish, phage therapy presents a promising alternative for controlling *V. parahaemolyticus* contamination while maintaining ecological balance in the aquaculture industry [42]. The diverse characteristics of the four phages identified in this study highlight the importance of continued exploration of phage diversity and host interactions to optimize their use in aquaculture-based decontamination strategies.

Additional analysis to test the effectiveness of the phages in clearing *V. parahaemolyticus* from contaminated oysters, as well as their impact on the microbiome and probiotic strains, is warranted. This will necessitate assessing the stability of these phages under varying salinity, temperature, and pH conditions and will provide insights into their resilience in oyster farming environments. Investigating whether these phages can enter oyster tissues and effectively reach *V. parahaemolyticus* populations residing within the oyster intestinal tract would also be important to determine their real-world application potential. Future efforts to isolate additional broad-host range phages might help identify the means to decontaminate multiple pathogenic strains.

## Supporting information

S1 Fig Lysis patterns of the CREW isolated phage. Image shows CW as a representative zone of clearing created by CREW when oyster homogenate is spotted on a VP strain G12408 seeded agar plate.
(TIF)

S2 Fig Lysis patterns of the SMY and OD isolated phages. Image shows SMY (SMILEY) and OD (OYD) representative zones of clearing created when the oyster homogenate is spotted on a VP strain G12408 seeded agar plate. They both produced a halo around the zone. This is indicative of either a possible phage with a lysogenic phase or the release of depolymerase enzymes from the lysed cells that are then diffusing through the agar to affect the growth of surrounding bacteria.
(TIF)

S3 Fig Plaque assay for REJA isolation. Shows the initial double layer agar overlay used to isolate REJA. The smallest plaques were isolated for further characterizations in the attempt to isolate a larger phage with broader host range.
(TIF)
